# Taxane-induced sensory peripheral neuropathy is associated with an *SCN9A* single nucleotide polymorphism in Japanese patients

**DOI:** 10.1186/s12885-020-06834-0

**Published:** 2020-04-16

**Authors:** Yuko Tanabe, Seiji Shiraishi, Kenji Hashimoto, Kazutaka Ikeda, Daisuke Nishizawa, Junko Hasegawa, Akihiko Shimomura, Yukinori Ozaki, Nobuko Tamura, Mayu Yunokawa, Kan Yonemori, Toshimi Takano, Hidetaka Kawabata, Kenji Tamura, Yasuhiro Fujiwara, Chikako Shimizu

**Affiliations:** 1 Department of Breast and Medical Oncology, National Cancer Center Hospital, 5-1-1 Tsukiji, Chuo-ku, Tokyo, 104-0045 Japan; 2 Department of Medical Oncology, Toranomon Hospital, 2-2-2 Toranomon, Minato-ku, Tokyo, 105-8470 Japan; 3 Department of Anesthesiology, Kohnodai Hospital, National Center for Global Health and Medicine, 1-7-1, Kohnodai, Ichikawa-shi, Chiba, 272-8516 Japan; 4 Addictive Substance Project, Tokyo Metropolitan Institute of Medical Science, 2-1-6 Kamikitazawa, Setagaya-ku, Tokyo, 156-8506 Japan; 5 Department of Breast and Endocrine Surgery, Toranomon Hospital, 2-2-2 Toranomon, Minato-ku, Tokyo, 105-8470 Japan; 6 Department of Breast Medical Oncology, Comprehensive Cancer Center, National Center for Global Health and Medicine, 1-21-1 Toyama, Shinjuku-ku, Tokyo, 162-8655 Japan

**Keywords:** Breast and ovarian cancer, rs13017637, *SCN9A*, *SCN10A*, Taxane-induced peripheral neuropathy

## Abstract

**Background:**

Sodium channels located in the dorsal root ganglion, particularly Nav1.7 and Nav1.8, encoded by *SCN9A* and *SCN10A*, respectively, act as molecular gatekeepers for pain detection. Our aim was to determine the association between TIPN and *SCN9A* and *SCN10A* polymorphisms*.*

**Methods:**

Three single nucleotide polymorphisms (SNPs) in *SCN9A* and two in *SCN10A* were investigated using whole-genome genotyping data from 186 Japanese breast or ovarian cancer patients classified into two groups as follows: cases that developed taxane-induced grade 2–3 neuropathy (*N* = 108) and controls (*N* = 78) with grade 0–1 neuropathy. Multiple logistic regression analyses were conducted to evaluate associations between TIPN and SNP genotypes.

**Results:**

*SCN9A-*rs13017637 was a significant predictor of grade 2 or higher TIPN (odds ratio (OR) = 3.463; *P* = 0.0050) after correction for multiple comparisons, and precision was improved when only breast cancer patients were included (OR 5.053, *P* = 0.0029). Moreover, rs13017637 was a significant predictor of grade 2 or higher TIPN 1 year after treatment (OR 3.906, *P* = 0.037), indicating its contribution to TIPN duration.

**Conclusion:**

*SCN9A* rs13017637 was associated with the severity and duration of TIPN. These findings are highly exploratory and require replication and validation prior to any consideration of clinical use.

## Background

Taxanes, such as paclitaxel and docetaxel, are used to treat a variety of tumor, especially breast and ovarian cancer [1, 2]. The common non-hematologic toxicity associated with these drugs is peripheral neuropathy. Taxane-induced peripheral neuropathy (TIPN) can be dose limiting, which would affect treatment efficacy, and can worsen quality of life. As susceptibility to TIPN differs greatly among patients [3, 4], predicting patient risk could improve therapeutic efficacy and quality of life by personalizing treatment.

To date, several genes have been reported as potential risk factors for TIPN, including those associated with transport (e.g. *ABCB1*) [5–7], metabolism (e.g. *CYP3A4*, *CYP3A5*, and *CYP2C8*) [5, 8–10], development and regeneration in the nervous system (e.g. *EPHA4* and *EPHA5*) [11], myelination in peripheral nerve (*FGD4*) [11], and β-tubulin (*TUBB2A*) [12]. Thus, many pharmacogenetic studies have attempted to identify polymorphisms that contribute to variations in susceptibility to TIPN; however, current data are insufficient to make genetic testings routine for these variants.

Many different hypotheses regarding the mechanisms of sensory TIPN have been generated, including excessive microtubule assembly, which causes the abnormal accumulation of disorganized microtubules disrupting normal cellular activities such as axonal transport and Schwann cell function; however, this process is still unclear [13, 14]. Voltage-gated sodium channels (VGSCs) located in the dorsal root ganglion (DRG), particularly Nav1.7 and Nav1.8, encoded by *SCN9A* and *SCN10A*, respectively, have important roles in the pathogenesis of human neuropathic pain [15–17]. VGSC mutations were reported to be associated with diseases of both the central and peripheral nervous systems [18]. Especially, mutations in *SCN9A* play a significant role in nociception signaling and have been associated with channelopathy-associated insensitivity to pain and paroxysmal extreme pain disorder, whereas the protein encoded by *SCN10A* is a tetrodotoxin-resistant sodium channel (SCNA) subunit that might be involved in painful peripheral neuropathy (PN) [15, 16]. Moreover, dorsal ganglionic Nav1.7 is upregulated in rats and humans administered paclitaxel. Blocking the function of Nav1.7 was found to partially attenuate paclitaxel-induced hyperalgesia in rats. This suggests that this protein is involved in paclitaxel-induced neuropathic pain [19, 20]. However, no pharmacogenetic studies have focused on the association between TIPN and SCNA in a clinical setting. Therefore, we investigated the effects of SNPs in genes encoding VGSC using a prospective cohort of well-characterized breast or ovarian cancer patients treated with taxanes.

## Methods

### Patients

Japanese women with breast or ovarian cancer receiving taxane regimens were enrolled in a prospective observational study to evaluate taxane-related toxicity and to explore gene variants associated with TIPN by performing a genome wide association study (UMIN000005294) [7]. The eligibility criteria are shown in previous study [7]. All patients provided written informed consent for adjuvant treatment and DNA collection for genetic analysis. The study was conducted in accordance with the Declaration of Helsinki and was approved by the local institutional review board (protocol number: 2013–199).

### Treatment

Breast cancer patients were treated with 4 cycles of cyclophosphamide (600 mg/m^2^) and doxorubicin (60 mg/m^2^) (AC) or cyclophosphamide (500 mg/m^2^) with epirubicin (100 mg/m^2^) and 5-fluorouracil (500 mg/m^2^) (CEF) every 3 weeks. Subsequently, paclitaxel (80 mg/m^2^, weekly) or docetaxel (75 mg/m^2^, every 3 weeks) was administered for 12 weeks. In addition, 4 cycles of cyclophosphamide (600 mg/m^2^, day1) and docetaxel (75 mg/m^2^, day1) every 3 weeks (CPA + DTX) was also an option for breast cancer treatment. Patients positive for HER2 by immunohistochemistry or gene amplification analysis received trastuzumab concurrently with paclitaxel (weekly or every 3 weeks). Ovarian cancer patients were treated with 6 cycles of paclitaxel (175 mg/m^2^) and carboplatin (AUC6, day 1) every 3 weeks (Tri TC), or paclitaxel (80 mg/m^2^, day 1, 8, 15) and carboplatin (AUC6, day 1) every 3 weeks (dose-dense TC).

### TIPN evaluation

Neuropathy was prospectively evaluated at baseline before paclitaxel treatment, at week 7, within 3 weeks at or after the final dose, and 1 year after the last dose of paclitaxel, based on the National Cancer Institute Common Terminology Criteria for Adverse Events 4.0 [21]. Evaluators of PN are five medical oncologists who are trained in toxicity assessment. One patient was evaluated by the same physician.

### Genotyping

A 10-mL blood sample was collected from each patient upon enrollment. Genotyping assays were performed for eligible patients with sufficient DNA in the sample. DNA was extracted from whole-blood samples using standard procedures. The extracted DNA was dissolved in TE buffer (10 mM tris-HCl and 1 mM EDTA, pH 8.0) and the concentration was adjusted to 100 ng/ml for whole-genome genotyping using a NanoDrop ND-1000 Spectrophotometer (NanoDrop Technologies, Wilmington, DE, USA). To analyze polymorphisms within and around the *SCN9A* and *SCN10A* gene regions, whole-genome genotype data were used. Whole-genome genotyping was performed using the Infinium assay II and the iScan system (Illumina, San Diego, CA) according to the manufacturer’s instructions. The OmniExpressExome-8 v1.1 BeadChip was used to genotype entire samples. The BeadChip included a number of probes that was specific to copy number variation markers, but most were for SNP markers on human autosomes or sex chromosomes. Approximately 900,000 SNP markers were included in the BeadChip. After whole-genome genotyping, data were analyzed using GenomeStudio Genotyping module v1.6.3 (Illumina) to evaluate the quality of results, and genotype data for SNPs with *SCN9A* and *SCN10A* gene annotations were extracted. The analyzed SNPs were *SCN9As* (rs7607967, rs12994338, and rs13017637) and *SCN10A* (rs12632942 and rs6795970), which were evaluated in previous reports [17, 22–25].

### Statistical analysis

The primary objective was to evaluate the association between TIPN grade and *SCN9A* and *SCN10A* variants. The association between genetic variants and the risk of grade 2–3 TIPN was assessed by logistic regression analysis with adjustments for confounding clinical covariates. The dependent variable was maximum TIPN grade (grades 0–1 or 2–3), and the independent variables were type of cancer (breast cancer vs. ovarian cancer), age (< 60 vs. ≥ 60 years), TIPN latency, and genotype data for each SNP; alternatively, the dependent variable was TIPN grade 1 year after treatment, and the independent variables were maximum TIPN grade, TIPN duration, and genotype data for each SNP. TIPN duration is defined as time from onset of PN until disappearance of PN or at 1 year of treatment. We preliminarily planned to dichotomize age at 60 years and compare TIPN, referring to our previous study [3]. For the selected polymorphisms, we considered either an additive, dominant, or recessive model. Odds ratios (ORs) and their 95% confidence intervals (95% CIs) were used to estimate relative risk. *SCN9A* and *SCN10A* frequencies were assessed for concordance with expectations based on the Hardy-Weinberg equilibrium (HWE), using the chi-square test and Fisher’s exact test. Given the sample size of cases (108 patients with grade 2–3 TIPN) and controls (78 patients with grade 0–1 TIPN) and assuming a power of 85%, the level of significance was 0.1. Power calculations were performed using G*power software v3.1.9.2. All analyses were performed using the highest grade of treatment-related sensory peripheral neuropathy. Patients for whom taxane-based chemotherapy was discontinued due to adverse events, except neuropathy, were excluded from the analyses. Multiple comparisons were accounted for by using Bonferroni adjustment α = 0.05 (corrected alpha (α = 0.05/5 = 0.01)). A two-sided adjusted *P*-value of < 0.05 was considered statistically significant, and all analyses were performed using SAS software (version 9.2; SAS Institute, Cary, NC, USA).

## Results

### Patient characteristics

We analyzed 186 of 221 Japanese women enrolled between February 2011 and October 2013 at the two study sites. Thirty-five patients were excluded, including patients lost to follow-up (*N* = 16), patients not treated with taxane (*N* = 9), patients with a history of prior taxane use (*N* = 5), patients for whom chemotherapy was discontinued due to adverse events other than peripheral neuropathy (*N* = 1), and other reasons (*N* = 4). The demographic and clinical characteristics of patients are listed in Table 1. The median age was 52 years (range, 25–81 years). The number of breast and ovarian cancer patients was 135 and 51, respectively. One hundred and seventy-seven patients received paclitaxel treatment, whereas nine patients received docetaxel. Among 186 patients studied, for 38 (20%) and 16 (8%), the dose was reduced or treatment was terminated because of neuropathy or reasons other than neuropathy, respectively. Maximum grades of TIPN were 0 (*N* = 2, 1%), 1 (*N* = 76, 41%), 2 (*N* = 91, 49%), and 3 (*N* = 17, 9%).

**Table 1:** Patient characteristics

	All patients (*N* = 186)	Cases TIPN Gr 2–3 (*N* = 108)	Control TIPN Gr 0–1 (*N* = 78)
Cancer Type			
Breast ca. ^a^	135	67	68
Ovarian ca. ^a^	51	41	10
Age (year) median (range)	52 (25–81)	56 (25–81)	48 (27–73)
Chemotherapy regimen^b^			
AC or CEF f/b wPTX	126 (68%)	67 (62%)	59 (77%)
AC or CEF f/b DTX	5 (3%)	0	5 (6%)
CPA + DTX	4 (2%)	0	4 (5%)
Dose dense TC	39 (21%)	34 (32%)	5 (6%)
Tri TC	12 (6%)	7 (6%)	5 (6%)
Maximum grade of TIPN^c^			
0	2 (1%)	0 (0%)	2 (3%)
1	76 (41%)	0 (0%)	76 (97%)
2	91 (49%)	91 (84%)	0 (0%)
3	17 (9%)	17 (16%)	0 (0%)
Cumulative taxane dose (mg/m^2^) median (range)			
PTX	960 (560–1440) (*N* = 177)	960 (560–1440) (*N* = 108)	960 (688–1440) (*N* = 69)
DTX	300 (270–400) (*N* = 9)	–	300 (270–400) (*N* = 9)
Time to develop TIPN (days) median (range)	33 (7–79)	28 (7–79)	35 (7–77)
Duration of TIPN (days) median (range)	446 (0–1416)	455 (70–1395)	416 (0–1327)
Grade of TIPN at 1 year after taxane			
0	44 (24%)	10 (9%)	34 (44%)
1	74 (40%)	38 (35%)	36 (46%)
2	41 (22%)	41 (38%)	0 (0%)
3	2 (1%)	2 (2%)	0 (0%)
unknown	25 (13%)	17 (16%)	8 (10%)
Full dose administration of taxane	132 (71%)	63 (58%)	69 (88%)
Dose reduction or termination of taxane due to TIPN	38 (20%)	35 (32%)	3 (4%)
Dose reduction or termination due to other reasons	16 (8%)	10 (9%)	6 (7%)
*SCN9A* rs7607967			
GG	24 (13%)	15 (14%)	9 (11%)
GA	84 (45%)	43 (40%)	41 (53%)
AA	78 (42%)	50 (46%)	28 (36%)
*SCN9A* rs12994338			
TT	13 (7%)	9 (8%)	4 (5%)
TC	80 (43%)	46 (43%)	34 (44%)
CC	93 (50%)	53 (49%)	40 (51%)
*SCN9A* rs13017637			
TT	3 (2%)	1 (1%)	2 (3%)
TC	37 (20%)	16 (15%)	21 (27%)
CC	146 (78%)	91 (84%)	55 (70%)
*SCN10A* rs12632942			
GG	44 (24%)	28 (26%)	16 (20%)
GA	87 (47%)	49 (45%)	38 (49%)
AA	55 (29%)	31 (29%)	24 (31%)
*SCN10A* rs6795970			
AA	4 (2%)	3 (3%)	1 (1%)
AG	47 (25%)	25 (23%)	22 (28%)
GG	135 (73%)	80 (74%)	55 (71%)

^a^*Ca.* cancer, ^b^*AC* cyclophosphamide (600 mg/m^2^) and doxorubicin (60 mg/m^2^), *CEF* cyclophosphamide (500 mg/m^2^) with epirubicin (100 mg/m^2^) and 5-fluorouracil (500 mg/m^2^), *f/b* followed by, *PTX* paclitaxel, *DTX* docetaxel, *CPA + DTX* four cycles of cyclophosphamide (600 mg/m^2^, day 1) and docetaxel (75 mg/m^2^, day 1) every 3 weeks, *dose dense TC* paclitaxel (80 mg/m^2^, day 1, 8, 15) and carboplatin (AUC6, day 1) every 3 weeks, *Tri TC* six cycles of paclitaxel (175 mg/m^2^) and carboplatin (AUC6, day 1) every 3 weeks, ^c^*TIPN* taxane-induced peripheral neuropathy

*SCN9A* rs7607067, *SCN9A* rs12994338, *SCN9A* rs13017637, *SCN10A* rs12632942, and *SCN10A* rs6795970 represent polymorphisms

### Distribution of *SCN9A* and *SCN10A* genotypes

Genotypes in each SNP are listed in Table 1. Genotyping call rates by SNP and patient were estimated. We confirmed that both call rates (by SNP and patient) were > 0.95 and all SNPs and samples were included in the analyses. The distributions of the *SCN9A* (rs7607967, rs12994338, and rs13017637) and *SCN10A* (rs12632942 and rs6795970) polymorphisms were all in accordance with the HWE (data not shown).

### Association between SNPs and maximum TIPN grade

Results of dominant genetic model were reported in text and tables. Results of other models were not shown because they were not statistically significant. Of the five SNPs evaluated, *SCN9A-rs13017637* was significantly associated with grade 2 or higher TIPN in all patients (OR, 3.463, *P* = 0.0050; Table 2). In addition, *SCN9A-rs13017637* was significantly associated with grade 2 or higher TIPN among the 135 breast cancer patients (OR, 5.053, *P* = 0.0029; Table 2).

**Table 2:** SNPs correlate with severity of TIPN† (grade 0–1 vs. grade 2–3) in all patients and in breast cancer patients (subset analysis), and SNPs correlate with TIPN^a^ (grade 0–1 vs. grade 2–3) at 1 year after completion of taxane treatment in all patients

Gene	SNP	Odds ratio	Odds ratio (95% C.I. ^b^)	*P*-value
*SCN9A*	rs7607967	1.749	0.889–3.441	0.1054
	rs12994338	0.952	0.492–1.841	0.8836
	rs13017637	3.463	1.456–8.237	0.0050
*SCN10A*	rs12632942	1.024	0.498–8.237	0.9491
	rs6795970	0.949	0.450–1.998	0.8894
A subset analysis in Breast only				
*SCN9A*	rs7607967	1.822	0.864–3.842	0.1151
	rs12994338	0.913	0.442–1.888	0.8061
	rs13017637	5.053	1.743–14.641	0.0029
*SCN10A*	rs12632942	1.262	0.569–2.802	0.567
	rs6795970	1.135	0.515–2.501	0.7537
Analysis at 1 year after completion of taxane treatment in all patients				
*SCN9A*	rs7607967	0.735	0.313–1.728	0.4805
	rs12994338	0.952	0.418–2.167	0.9069
	rs13017637	3.906	1.084–14.075	0.03719
*SCN10A*	rs12632942	0.560	0.219–1.430	0.225
	rs6795970	1.834	0.697–4.826	0.2192

^a^*TIPN* taxane-induced peripheral neuropathy, ^b^*C.I.* confidence interval

### Association between SNPs and TIPN grade 1 year after taxane

Of the five SNPs evaluated, *SCN9A-rs13017637* was significantly associated with grade 2 or higher TIPN in all patients (OR, 3.906, *P* = 0.037; Table 2). However, no SNPs were significantly associated with grade 2 or higher TIPN among the 135 breast cancer patients.

## Discussion

We evaluated the effect of sodium channel-related genes on TIPN development in breast or ovarian cancer patients treated with taxane. Multiple logistic regression revealed that *SCN9A-rs13017637* was significantly associated with grade 2 or higher TIPN. It was also significantly associated with grade 2 or higher TIPN 1 year after the completion of taxane-based therapy. To our knowledge, this is the first study demonstrating that SNPs of sodium channel-related genes are associated with increased TIPN severity in Japanese patients treated with taxane.

Sodium channels in the DRG function as molecular gatekeepers of pain detection at peripheral nociceptors. Nine sodium channel subunits have been identified (Nav1.1–Nav1.9), each with a unique central and peripheral nervous system distribution. Gain-of-function (GOF) mutations in *SCN9A,* encoding Nav1.7, cause inherited erythromelalgia and paroxysmal extreme pain disorder, rare familial diseases associated with excruciating pain [26–28]. In contrast, loss-of-function mutations cause congenital pain insensitivity, a rare autosomal recessive disease [26, 27]. Rare missense variants have also been reported in *SCN9A* [29, 30] and *SCN10A* [30, 31] in patients with painful small fiber neuropathy. It is thought that enhanced Nav channel activity might directly contribute to pain experienced by these patients because several identified missense variants exerted GOF effects in cell-based electrophysiology assays [32]. Our findings indicate that *SCN9A-rs13017637* is a risk factor for TIPN severity. However, the function of *SCN9A-rs13017637* is not known. *SCN10A*-rs6795970 reportedly affects human pain sensitivity. A recent study suggested that SNPs in voltage-gated sodium channel genes (*SCN9A-*rs6754031 and *SCN10A*-rs12632942) can cause oxaliplatin-based peripheral neurotoxicity [24, 25, 33, 34]. A polymorphism in *SCN9A* (rs6746030) was also reported to be associated with decreased neurotoxicity [35]. However, these polymorphisms were not associated with TIPN in our study. Thus, there could be different predisposing polymorphisms for different ethnic groups. Moreover, a selective Nav1.7 channel blocker was shown to decrease firing frequency in the human DRG with spontaneous action potentials. This suggests that Nav1.7 might be a potential new target for TIPN treatment [19]. However, these results must be validated by larger and prospective studies.

There were some limitations to this study. First, we focused on paclitaxel or docetaxel as taxane regimens. There might be possible differences between paclitaxel and docetaxel regarding the severity or duration of PN. Moreover, platinum added to taxane might influence PN because it was reported as a risk factor for chemotherapy-induced PN [36]. Second, although cumulative drug doses were different, no adjustments were made for multiplicity of inferences. However, differences in cumulative doses were small and would be less likely to influence TIPN development. Third, our study focused on *SCN9A* and *SCN10A*, which were evaluated based on previous studies; other sodium channel subunits such as Nav1.9 were not evaluated. Fourth, there was a possible issue with type I error for this study because of multiple test.

## Conclusion

TIPN was associated with *SCN9A-rs13017637* in Japanese breast or ovarian cancer patients who received taxane. This finding is highly exploratory and requires replication and validation prior to any consideration of clinical use.

## Data Availability

The datasets used and analyzed during the current study are available from the corresponding author on reasonable request.

## Abbreviations

TIPN: Taxane Induced Peripheral Neuropathy
VGSCs: Voltage Gated Sodium Channels
DRG: Dorsal Root Ganglion
SCNA: Synuclein alpha
PN: Peripheral Neuropathy
AC: Doxorubicin and Cyclophosphamide
CEF: Cyclophosphamide and Epirubicin and 5-Fluorouracil
CPA: Cyclophosphamide
DTX: Docetaxel
TC: Carboplatin and Paclitaxel
ORs: Odds ratios
Cis: Confidence intervals
HWE: Hardy Weinberg Equilibrium
GOF: Gain Of Function
